# Effect of acarbose combined with diet intervention on glycolipid metabolism in patients with primary polycystic ovarian syndrome complicated with impaired glucose tolerance

**DOI:** 10.12669/pjms.38.4.4598

**Published:** 2022

**Authors:** Qian Yang, Wenjuan Zhang, Junfei Zhang, Shuai Niu

**Affiliations:** 1 Qian Yang, Department of Nutrition, Baoding First Central Hospital, Baoding, 071000, Hebei, China; 2 Wenjuan Zhang, Department of Quality Control, Baoding First Central Hospital, Baoding, 071000, Hebei, China; 3 Junfei Zhang, Department of Nutrition, Baoding First Central Hospital, Baoding, 071000, Hebei, China; 4 Shuai Niu, Department of Nutrition, Baoding First Central Hospital, Baoding, 071000, Hebei, China

**Keywords:** Acarbose, Diet intervention, Impaired glucose tolerance, Polycystic ovarian syndrome

## Abstract

**Objectives:**

To investigate the efficacy of acarbose combined with diet intervention in patients with primary polycystic ovarian syndrome (PCOS) complicated with impaired glucose tolerance (IGT) and its effect on their glycolipid metabolism.

**Methods:**

A total of 92 patients with primary PCOS complicated with IGT treated in our hospital from December 2018 to December 2020 were selected, and divided into two groups using a random number table. The control group received routine lifestyle intervention. On this basis, the observation group was treated with acarbose combined with diet intervention. The menstrual recovery rate, ovulation rate and pregnancy rate after treatment, as well as sex hormone levels, glycolipid metabolism and inflammatory factor levels before and after treatment were compared between the two groups.

**Results:**

After treatment, the menstrual recovery rate and ovulation rate of the observation group were significantly higher than those of the control group (*P* < 0.05). Among the patients with fertility needs in the two groups, the pregnancy rate of the observation group was significantly higher compared with the control group (*P* < 0.05). Before treatment, sex hormone levels showed no obvious differences between the two groups (*P* > 0.05). After treatment, all indicators of sex hormone were improved in both groups, and the improvement in the observation group was more obvious than that in the control group (*P* < 0.05). Before treatment, no obvious difference was found in glycolipid metabolism between the two groups (*P* > 0.05). After treatment, 2hPG, LDL-C and TG were improved in both groups, and the improvement was more significant in the observation group compared with the control group (*P* < 0.05). Before treatment, inflammatory factor levels were not significantly different between the two groups (*P* > 0.05). After treatment, inflammatory factor levels in both groups were improved, which was more obvious in the observation group than the control group (*P* < 0.05).

**Conclusions:**

The application of acarbose combined with diet intervention in patients with primary PCOS complicated with IGT can effectively enhance the efficacy, help patients increase ovulation rate and pregnancy rate, and improve sex hormone levels, glycolipid metabolism and inflammatory factor levels.

## INTRODUCTION

Polycystic ovary syndrome (PCOS) is a commonly occurring endocrine disorder characterized by hirsutism, anovulation, and polycystic ovaries.^1^ Often comorbid with insulin resistance, dyslipidemia, and obesity, it also carries significant risk for the development of cardiovascular and metabolic sequelae, including Type-2 diabetes mellitus (T2DM) and metabolic syndrome.^2^ PCOS occurs frequently in women of childbearing age and have an extremely serious impact on the daily life of patients.^3^

PCOS is characterised by hyperandrogenaemia, hyperinsulinaemia, and deranged adipokines secretion from the adipose tissue. In addition to decreased insulin sensitivity, women with PCOS exhibit beta cell dysfunction. Further metabolic complications lead to dyslipidaemia, worsening obesity and glucose tolerance, high prevalence of metabolic syndrome, and greater susceptibility to diabetes.^4^ Impaired glucose tolerance (IGT) is one of the complications of PCOS, which can cause pre-diabetes and a high risk of developing diabetes.^5^ Clinically, lifestyle intervention is always used for patients with primary PCOS combined with IGT, that is, the metabolic abnormalities of patients are improved through exercise and diet guidance. However, its efficacy is not ideal. Therefore, it is clinically attempted to treat patients with additional acarbose, which can reduce insulin resistance (IR), and diet intervention. By controlling the diet of patients, their glycolipid metabolism can be improved, thus enhancing the efficacy.^6^ In this study, 92 patients with primary PCOS complicated with IGT treated in our hospital from December 2018 to December 2020 were selected as subjects, to investigate the efficacy of acarbose combined with diet intervention in patients with primary PCOS complicated with IGT and its effect on their glycolipid metabolism. It is reported as follows.

## METHODS

A total of 92 patients with primary PCOS complicated with IGT treated in our hospital from December 2018 to December 2020 were selected and divided into two groups using a random number table. In the control group (n = 46), the patients aged from 22 to 31 years with an average age of (26.50 ± 4.50) years, weighed from 58 kg to 73 kg with an average weight of (65.50 ± 7.50) kg, and had a course of disease from 1 to 6 years with an average course of (3.50 ± 2.50) years. In the observation group (n = 46), the age range was 21-32 years (average, 26.50 ± 5.50 years), the weight range was 57 kg-72 kg (average, 64.50 ± 7.50 kg), and the range of course of disease was 1-6 years (average, 3.50 ± 2.50 years). Age, body weight and course of disease showed no obvious differences between the two groups, suggesting comparability. This study was approved by the medical ethics committee.

### Ethical Approval:

The study was approved by the Institutional Ethics Committee of Baoding First Central Hospital on May 21, 2017, with lot number 2017-28, and written informed consent was obtained from all participants.

### Inclusion criteria:

- Patients with obesity and abnormal menstruation, and clinically diagnosed with primary PCOS^7^;
- Patients with fasting plasma glucose (FPG) < 6.1 mmol/L, and two-hour postprandial blood glucose (2hPG) of 7.8 mmol/L-11.1 mmol/L after glucose tolerance test;
- Patients who were informed of this study and signed the informed consent.

### Exclusion criteria:

- Patients recently receiving relevant drug therapies;
- Women in pregnancy or lactation;
- Ppatients with allergic reactions to the drugs used in this study;
- Patients with hyperandrogenemia or ovulation disorders caused by the thyroid and adrenal glands;
- Patients with severe organ dysfunction;
- Patients who had mental illness and could not cooperate with this study.

The control group received routine lifestyle intervention, mainly including psychological nursing, exercise and diet guidance. On this basis, the observation group was additionally treated with acarbose combined with diet intervention. The initial dose of Acarbose Tablets (Hangzhou Zhongmei Huadong Pharmaceutical Co., Ltd., Guoyao Zhunzi: H20020202, specification: 50 mg * 30 s) was 50 mg/time, 3 times/d. The whole tablet was suggested to be swallowed or chewed with food. According to the actual condition of the patients, the dose was increased to 0.1 g/time, 3 times/d by the attending physician. The course of treatment was six month. The main contents of diet intervention included:

### Comprehensive examination:

The examination items mainly included body mass, blood glucose, blood lipid, liver function and sex hormone. After understanding the patients’ condition, the medical staff designed a scientific and reasonable diet plan for the patients. The patients needed to eat more high-protein and high-fiber food, and avoided eating high-calorie, high-fat and high-sugar food. In the diet plan, the proportion of fruits and vegetables could be increased and the intake of meat and viscera could be reduced.

### Health education:

The medical staff has remained engaged in active communication with the patient, made the patient aware of the importance of correct diet for treatment, explained the meaning of food choices in the meal plan, and adjusted the meal plan to suit the patients’ personal preferences. *Adjustment of diet plan:* During treatment, the medical staff closely monitored the changes in the patients’ condition, proactively communicated with the patient, helped the patient relieve psychological pressure, increased treatment confidence, and adjusted the diet plan reasonably according to the changes in the patients’ condition and recovery.

### Observation indicators:

(1) Menstrual recovery rate, ovulation rate and pregnancy rate in patients were compared between the two groups. Among them, the recovery rate of menstruation refers to the percentage of the number of patients who return to normal menstruation after treatment of menstrual disorder, amenorrhea, too little menstrual blood or functional uterine bleeding. The ovulation cycle was assessed based on the patients’ menstrual cycle. Patients were instructed to use an ovulation strip to measure urinary luteinizing hormone levels during ovulation to assess ovulation.

Sex hormone levels, including serum estradiol (E2), luteinizing hormone (LH), follicle stimulating hormone (FSH) and testosterone (T), were compared between the two groups before and after treatment.

Glycolipid metabolism including FPG, 2hPG, low-density lipoprotein cholesterol (LDL-C) and triglyceride (TG), were compared between the two groups before and after treatment.Serum inflammatory factor levels, including C-reactive protein (CRP), interleukin-6 (IL-6) and tumor necrosis factor-alpha (TNF-α), were compared between the two groups before and after treatment.

### Statistical Methods:

All data were analyzed using SPSS22.0. The enumeration data were expressed as percentage (%) and compared using the test. *P* < 0.05 was considered as statistically significant.

## RESULTS

**Fig.1:**
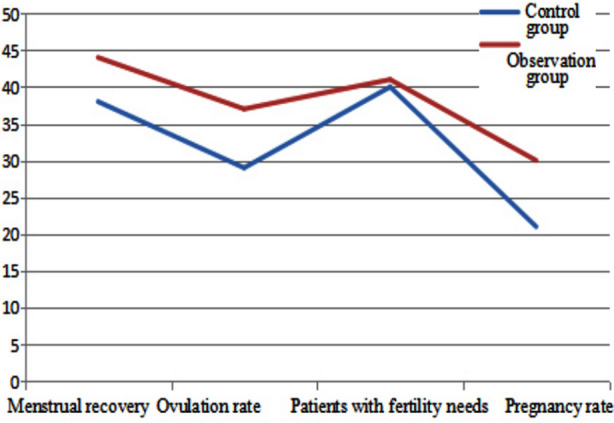
Comparison in efficacy between two groups.

After treatment, the menstrual recovery rate and ovulation rate of the observation group were significantly higher than those of the control group (*P* < 0.05). The pregnancy rate of the observation group was significantly higher compared with the control group (*P* < 0.05), Table-I and Fig.1.Before treatment, sex hormone levels showed no obvious differences between the two groups (*P* > 0.05). After treatment, all indicators of sex hormone were improved in both groups, and the improvement in the observation group was more obvious than that in the control group (*P* < 0.05), Table-II.

**Table-I:** Comparison in efficacy between two groups [n (%)].

Group	N	Menstrual recovery	Ovulation rate	Patients with fertility needs	Pregnancy rate
Control group	46	38 (82.61%)	29 (63.04%)	40	21 (52.50%)
Observation group	46	44 (95.65%)	37 (80.43%)	41	30 (73.17%)
x^2^		8.776	7.457		9.148
P		0.003	0.006		0.002

**Table-II:** Comparison in sex hormone levels between two groups before and after treatment (± s).

Group	N	LH (U/L)		E2 (pmol/L)		FSH (U/L)		T (ng/mL)	

		Before treatment	After treatment	Before treatment	After treatment	Before treatment	After treatment	Before treatment	After treatment
Control group	46	16.22 ± 3.64	8.43 ± 2.05*	152.34 ± 19.83	133.27 ± 15.51*	5.86 ± 1.68	4.14 ± 1.31*	0.62 ± 0.07	0.32 ± 0.09*
Observation group	46	16.45 ± 3.74	5.34 ± 1.25*	151.45 ± 19.54	112.13 ± 13.62*	5.91 ± 1.42	3.06 ± 0.97*	0.61 ± 0.06	0.25 ± 0.07*
*t*		0.299	8.729	0.217	6.946	0.154	4.494	0.736	4.164
*P*		0.766	0.000	0.829	0.000	0.878	0.000	0.464	0.000

Notes:

* indicates comparison with the same group before treatment (P < 0.05).

Before treatment, no obvious difference was found in glycolipid metabolism between the two groups (*P* > 0.05). After treatment, 2hPG, LDL-C and TG were all improved in both groups, and the improvement was more significant in the observation group compared with the control group (*P* < 0.05) (Table-III).Before treatment, inflammatory factor levels were not significantly different between the two groups (*P* > 0.05). After treatment, inflammatory factor levels in both groups were improved significantly, which was more obvious in the observation group than the control group (*P* < 0.05), Table-IV.

**Table-III:** Comparison in glycolipid metabolism between two groups before and after treatment [( ± s), mmol/L].

Group	N	FPG		2hPG		LDL-C		TG	

		Before treatment	After treatment	Before treatment	After treatment	Before treatment	After treatment	Before treatment	After treatment
Control group	46	5.18 ± 0.55	5.22 ± 0.61	8.98 ± 1.79	7.79 ± 1.35*	3.36 ± 0.25	2.79 ± 0.21*	1.61 ± 0.29	1.28 ± 0.33*
Observation group	46	5.21 ± 0.63	5.24 ± 0.59	8.86 ± 1.74	7.23 ± 1.01*	3.37 ± 0.22	1.92 ± 0.22*	1.59 ± 0.26	1.15 ± 0.24*
t		0.243	0.160	0.326	2.253	0.204	19.401	0.348	2.161
P		0.808	0.873	0.745	0.027	0.839	0.000	0.729	0.033

Notes:

* indicates comparison with the same group before treatment (P < 0.05).

**Table-IV:** Comparison in inflammatory factor levels between two groups before and after treatment ( ± s).

Group	N	CRP (mg/L)		IL-6 (pg/ml)		TNF-α (pg/ml)	

		Before treatment	After treatment	Before treatment	After treatment	Before treatment	After treatment
Control group	46	5.68 ± 1.96	3.34 ± 1.72*	28.48 ± 5.52	21.78 ± 4.59*	29.38 ± 5.13	19.25 ± 5.34*
Observation group	46	5.83 ± 1.83	2.16 ± 0.61*	28.73 ± 4.61	17.30 ± 4.25*	29.57 ± 4.23	13.54 ± 2.54*
t		0.379	4.385	0.236	4.857	0.194	6.549
P		0.705	0.000	0.814	0.000	0.847	0.000

Notes:

* indicates comparison with the same group before treatment (P < 0.05).

## DISCUSSION

As the most common endocrine disease in women, PCOS will lead to amenorrhea, obesity, hirsutism and infertility, which will not only affect the daily life of patients, but also cause huge psychological pressure.^8^ The metabolic abnormalities in PCOS patients are mainly manifested as hyperinsulinemia, dyslipidemia and systemic inflammation caused by IR, always accompanied by T2DM and hypertension, which are common complications in PCOS patients.^9^ The occurrence of diabetes will go through the stage of IGT, during which the blood glucose level of patients is between the normal level and the diagnostic level of diabetes, also known as pre-diabetes or high-risk diabetes.^10^,^11^ Clinically, the treatment of patients with primary PCOS combined with IGT is mainly based on routine lifestyle intervention, which helps patients gradually improve their metabolic abnormalities through exercise.^12^ However, its efficacy is poor, and its improvement effects on sex hormone, glycolipid metabolism and inflammatory factors are not ideal. In view of this situation, it is clinically attempted to treat patients with additional acarbose combined with diet intervention on this basis.^13^,^14^ Acarbose is an α-glucosidase inhibitor, which can inhibit the activity of α-glucosidases such as maltase and glucoamylase, and slow down the starch decomposition into glucose and sucrose decomposition into glucose, thus reducing the absorption of glucose in the intestine, relieving postprandial hyperglycemia, and achieving the decline in blood glucose in patients.^15^,^16^ In addition, diet intervention is a nursing intervention for patients’ dietary habits. By adjusting the dietary structure and reducing the intake of high-sugar and high-calorie food, it can help patients control their body weight, improve their physical quality and control their endocrine, so as to improve the quality of life of patients and reduce the influence of obesity on the disease.^17^,^18^

Our results showed that after treatment, the menstrual recovery rate, ovulation rate and pregnancy rate of the observation group were significantly higher than those in the control group (*P* < 0.05). Previous studies have shown that acarbose combined with clomiphene is more effective than metformin in inducing ovulation and reducing body mass index in infertile women with polycystic ovary syndrome.^19^ Before treatment, no obvious differences were found in sex hormone levels, glycolipid metabolism or inflammatory factor levels between the two groups (*P* > 0.05). After treatment, all ex-hormone levels, glycolipid metabolism and inflammatory factor levels were improved in both groups, which were more significant in the observation group compared with the control group (*P* < 0.05). Acarbose has been reported to improve hirsutism, acne, and menstrual irregularities by reducing androgen concentration and increasing androgen binding. In addition, obese women with PCOS experienced significant weight loss and improvement in cardiovascular risk markers six months after acarbose treatment.^20^

Further analysis reveals that acarbose has the effect of slowing down the intestinal absorption of glucose, which can effectively improve the blood glucose level of patients. Additionally, diet intervention mainly develops a scientific and reasonable diet plan for patients, to reduce the intake of high-sugar and high-calorie food and inhibit fat synthesis in patients, and thereby regulating the lipid metabolism and improving the endocrine disorders of patients. Moreover, the patients’ body weight is controlled, which can effectively reduce the influence of aggravated inflammatory state caused by obesity and improve their fertility, thus increasing the menstrual recovery rate, ovulation rate and pregnancy rate.

### Limitations of this study:

The number of subjects included in this study is limited. Given that the study was a small randomized controlled trial, these results are not conclusive. Further large-scale trials are needed to confirm these results.

## CONCLUSIONS

The application of acarbose combined with diet intervention in patients with primary PCOS complicated with IGT can effectively enhance the efficacy, help patients increase ovulation rate and pregnancy rate, and improve sex hormone levels, glycolipid metabolism and inflammatory factor levels.

### Authors’ Contributions:

**QY** & **WZ:** Designed this study. prepared this manuscript, are responsible and accountable for the accuracy or integrity of the work.

**JZ:** Collected and analyzed clinical data.

**SN:** Significantly revised this manuscript.
